# Nobiletin, a hexamethoxyflavonoid from citrus pomace, attenuates G1 cell cycle arrest and apoptosis in hypoxia-induced human trophoblast cells of JEG-3 and BeWo via regulating the p53 signaling pathway

**DOI:** 10.29219/fnr.v65.5649

**Published:** 2021-09-24

**Authors:** Mengling Zhang, Jian Liu, Rui Zhang, Zengenni Liang, Shenghua Ding, Huanling Yu, Yang Shan

**Affiliations:** 1 Longping Branch Graduate School, Hunan University, Changsha, Hunan Province, China; 2 School of Public Health, Beijing Key Laboratory of Environmental Toxicology, Capital Medical University, Beijing, China; 3 Hunan Agriculture Product Processing Institute, Hunan Academy of Agricultural Sciences, Changsha, Hunan Province, China; 4 Hunan Key Lab of Fruits & Vegetables Storage, Processing, Quality and Safety, Hunan Agricultural Products Processing Institute, Changsha, Hunan Province, China; 5 School of Medical Humanity, Peking University, Beijing, China

**Keywords:** Nobiletin, p53, molecular docking, molecular dynamics, spectrum, apoptosis, trophoblast cells

## Abstract

**Background:**

Hypoxia is associated with abnormal cell apoptosis in trophoblast cells, which causes fetal growth restriction and related placental pathologies. Few effective methods for the prevention and treatment of placenta-related diseases exist. Natural products and functional foods have always been a rich source of potential anti-apoptotic drugs. Nobiletin (NOB), a hexamethoxyflavonoid derived from the citrus pomace, shows an anti-apoptotic activity, which is a non-toxic constituent of dietary phytochemicals approved by the Food and Drug Administration. However, their effects on hypoxia-induced human trophoblast cells have not been fully studied.

**Objective:**

The aim of this study was to investigate the protective effects of NOB on hypoxia-induced apoptosis of human trophoblast JEG-3 and BeWo cells, and their underlying mechanisms.

**Design:**

First, the protective effect of NOB on hypoxia-induced apoptosis of JEG-3 and BeWo cells was studied. Cell viability and membrane integrity were determined by CCK-8 assay and lactate dehydrogenase activity, respectively. Real Time Quantitative PCR (RT-qPCR) and Western blot analysis were used to detect the mRNA and protein levels of HIF1α. Propidium iodide (PI)-labeled flow cytometry was used to detect cell cycle distribution. Cell apoptosis was detected by flow cytometry with Annexin V-FITC and PI double staining, and the expression of apoptosis marker protein cl-PARP was detected by Western blot analysis. Then, the molecular mechanism of NOB against apoptosis was investigated. Computer molecular docking and dynamics were used to simulate the interaction between NOB and p53 protein, and this interaction was verified *in vitro* by Ultraviolet and visible spectrum (UV-visible spectroscopy), fluorescence spectroscopy and circular dichroism. Furthermore, the changes in the expression of p53 signaling pathway genes and proteins were detected by RT-qPCR and Western blot analysis, respectively.

**Results:**

Hypoxia treatment resulted in a decreased cell viability and cell membrane integrity in JEG-3 and BeWo cell lines, and an increased expression of HIF1α, cell cycle arrest in the G1 phase, and massive cell apoptosis, which were alleviated after NOB treatment. Molecular docking and dynamics simulations found that NOB spontaneously bonded to human p53 protein, leading to the change of protein conformation. The intermolecular interaction between NOB and human p53 protein was further confirmed by UV-visible spectroscopy, fluorescence spectroscopy and circular dichroism. After the treatment of 100 μM NOB, a down-regulation of mRNA and protein levels of p53 and p21 and an up-regulation of BCL2/BAX mRNA and protein ratio were observed in JEG-3 cells; however, there was also a down-regulation of mRNA and protein levels observed for p53 and p21 in BeWo cells after the treatment of NOB. The BCL2/BAX ratio of BeWo cells did not change after the treatment of 100 μM NOB.

**Conclusion:**

NOB attenuated hypoxia-induced apoptosis in JEG-3 and BeWo cell lines and might be a potential functional ingredient to prevent pregnancy-related diseases caused by hypoxia-induced apoptosis. These findings would also suggest the exploration and utilization of citrus resources, and the development of citrus industry.

## Popular scientific summary

- Nobiletin reduced cell cycle arrest in the G1 phase, improved cell viability and cell membrane integrity of JEG-3 and BeWo cell lines caused by hypoxia.
- This study indicated that nobiletin attenuated hypoxia-induced apoptosis in cultured human choriocarcinoma trophoblast cell lines of JEG-3 and BeWo by regulating the p53 signaling pathway.
- Nobiletin spontaneously bonds to human p53 protein, leading to the change of protein conformation of p53, which was confirmed by molecular docking and dynamics, UV-visible spectroscopy, fluorescence spectroscopy and circular dichroism.

Hypoxia is a common risk factor for placenta-mediated pregnancy complications, such as preeclampsia, fetal growth restriction, preterm birth, and stillbirth. Hypoxia-induced apoptosis of trophoblast cells is an important mechanism of these diseases (4). On one hand, the apoptosis of trophoblast cells could undermine the invasion and migration of extravillous cytotrophoblasts (evCTBs), thereby badly impacting the ability of evCTBs differentiating into trophoblast giant cells, which is detrimental to embryo implantation and early pregnancy maintenance (5). On the other hand, hypoxia leads to insufficient differentiation of cytotrophoblasts (CTBs) into syncytiotrophoblasts (STBs), thus affecting the endocrine, protection and migration functions of the placenta (6). Hence, the apoptosis of trophoblast cells might impair the placental function, thereby threatening the health of the mother and fetus.

Citrus is one of the fruits with the highest yield in the world (7). According to previous studies, nobiletin (5,6,7,8,3’,4’-hexamethoxyflavone, NOB), a highly methoxylated flavonoid, is one of the by-products in citrus processing, with anti-apoptotic activity. Propofol-induced neuronal apoptosis was attenuated by NOB in ischemic brain injury in male Sprague-Dawley rats (8). Myocyte apoptosis in cardiac hypertrophy was inhibited by treatment with NOB in male C57BL/6 mice (9). Hepatocyte apoptosis after liver transplantation in rats was reduced by NOB (10). Meanwhile, NOB is a non-toxic constituent of dietary phytochemicals approved by the Food and Drug Administration (11). In addition, this research study found that NOB was safe for human choriocarcinoma trophoblast cells (BeWo cells) (12). Based on its anti-apoptotic properties and safety considerations, NOB was hypothesized as a potential phytochemical that might protect the trophoblast cells from hypoxia-induced apoptosis.

JEG-3 and BeWo cells are usually used as trophoblast models. JEG-3 cells are invasive and migratory (13), while BeWo cells could differentiate into STBs (14). In this study, JEG-3 and BeWo cell models were used to evaluate the effect of NOB on the apoptosis of hypoxia-induced trophoblast cells. On this basis, the protective effect of NOB in reducing hypoxia-induced apoptosis was explored through the molecular docking and dynamics simulation of NOB and p53 protein, which is verified *in vitro* by UV-visible spectroscopy, fluorescence spectroscopy and circular dichroism, and the mRNA and protein expressions of the p53 signaling pathway.

## Materials and methods

### Materials

NOB (>95.0% purity) and cobalt chloride hexahydrate were purchased from Aladdin Shanghai Biochemical Technology (Shanghai, China). Recombinant human p53 protein was obtained from Boston Biochem (Beijing, China). F12 (Ham) medium, Dulbecco’s modification of Eagle’s medium Dulbecco (DMEM) (high glucose), and dimethyl sulfoxide (DMSO) were obtained from Boster Biological Technology (Pleasanton, CA, USA). Fetal bovine serum (FBS) and Dulbecco’s phosphate-buffered saline were obtained from Corning (New York, NY, USA). Trypsin-Ethylene diamine tetraacetic acid (EDTA) solution, trypsin without EDTA, penicillin, and streptomycin were purchased from Keygen (Beijing, China). JEG-3 and BeWo cells were purchased from Peking Union Medical College (Beijing, China).

### In vitro cell culture

JEG-3 cells were seeded at a density of 1 × 10^5^ cells/mL in sterile culture flasks, were cultured in DMEM (high glucose) with 10% (v/v) FBS and 100 μg/mL penicillin/streptomycin, and incubated in an incubator containing 95% air and 5% carbon dioxide at 37°C. Seeded at a density of 1 × 10^5^ cells/mL in sterile culture flasks, BeWo cells were cultured in F12 (Ham) medium with 15% (v/v) FBS and 100 μg/mL penicillin/streptomycin, and incubated in an incubator containing 95% air and 5% carbon dioxide at 37°C. Reaching the density of 80%, the cells (JEG-3 and BeWo) were passaged using trypsin-EDTA solution and sub-cultured in new flasks. Cell culture media were replaced every 3 days for BeWo cells and every 2 days for JEG-3 cells. As for JEG-3 cells, NOB and cobalt chloride were, respectively, dissolved in DMEM (high glucose) containing 0.1% DMSO. The DMEM (high glucose) in the control group only contained DMSO (0.1%). With regard to BeWo cells, NOB and cobalt chloride were, respectively, dissolved in F12 (Ham) medium containing 0.1% DMSO. The F12 (Ham) medium in the control group only contained DMSO (0.1%).

### Hypoxia model

In this research study, cobalt chloride was used to establish a hypoxic model of JEG-3 and BeWo cells. JEG-3 cells were seeded at a density of 1 × 10^5^ cells/mL and cultured for 48 h as described above (see section ‘*In vitro* cell culture’). BeWo cells were seeded at a density of 1 × 10^5^ cells/mL and cultured for 72 h as described above (see section ‘*In vitro* cell culture’). After removing the media, the cells were cultured in serum-free medium supplied with 500 μM cobalt chloride and NOB at different concentrations (0, 10, 33, and 100 μM). Cells treated without cobalt chloride and NOB was considered as the control group. The cells were collected after another 12 h of culture for the subsequent detections.

### Cell viability assay

A CCK-8 kit (MultiSciences, Hangzhou, China) was used to determine the viability of cells, according to the manufacturer’s recommendations. After hypoxia treatments (see section ‘Hypoxia model’), CCK-8 reagent (10 μL) was added to each well of 96-well plates and incubated at 37°C for another 4 h. The absorbance was measured with a microplate reader (BioTek, Winooski, VT, USA) at a wavelength of 450 nm.

### Lactate dehydrogenase activity assay

After hypoxia treatment (see section ‘Hypoxia model’), the cells were centrifuged for 5 min at 1,500 rpm, and the medium was collected for testing lactate dehydrogenase (LDH) activity. LDH activity was tested using a LDH kit (Jiancheng, Nanjing, China) according to the manufacturer’s recommendations. Data were expressed as the percentage release of LDH from the treated cells by comparing it with the control group.

### Quantitative RT-PCR analysis

The mRNA expression levels were determined using previously reported methods with minor modifications (15). PCR was conducted at 95°C for 4 min, followed by 39 cycles for 10 s at 95°C and 45 s at 60°C. Human gene-specific primers were used as shown in Table 1.

**Table 1:** Primer sequences used in this study.

Gene	Forward primer	Reverse primer
*HIF1α*	GGCGCGAACGACAAGAAAAAG	CCTTATCAAGATGCGAACTCACA
*p53*	AAAAGTCTAGAGCCACCGTCC	AGTCTGGCCAATCCAGGGAAG
*MDM2*	TCTCCTGCCTCAGCCTTCCAAG	GCCAGGTGCCTCACATCTGTAATC
*p21*	CCTGTCACTGTCTTGTACCCT	GCGTTTGGAGTGGTAGAAATCT
*BCL2*	GATAACGGAGGCTGGGATGC	TCACTTGTGGCCCAGATAGG
*BAX*	AAACTGGTGCTCAAGGCCC	CTTCAGTGACTCGGCCAGG
*Actb*	TCCTTCCGCAGCTATTTATGAT	CACAGTATAGGATGGTCTGGAC

### Western blot analysis

The levels of protein expression were measured using previously reported methods (12). Total proteins from the treated JEG-3 and BeWo cells were isolated using Radio-Immunoprecipitation assay (RIPA) buffer with 1% Phenylmethanesulfonyl fluoride (PMSF) (Abcam, Cambridge, UK). The protein concentrations were quantified using the Bicinchoninic acid (BCA) protein assay kit (Keygen, Beijing, China). The protein levels of the target genes were determined by Western blot analysis. The working concentration of primary anti-β-actin (ACTB) (Abcam, Cambridge, UK) was 1:2,000. The working concentrations of primary anti-cleaved Poly ADP-ribose polymerase (PARP) (Abcam, Cambridge, UK), anti-p21 (Abcam, Cambridge, UK), anti-MDM2 (Abcam, Cambridge, UK), anti-BCL2 (Abcam, Cambridge, UK), and anti-BAX (Abcam, Cambridge, UK) were 1:1,000. The working concentrations of primary anti-HIF1*α* (Abcam, Cambridge, UK) and anti-p53 antibody (Abcam, Cambridge, UK) were 5 μg/mL. The relative amount of each target protein was normalized to ACTB.

### Cell cycle analysis

Cell cycle distribution was detected using a cell cycle detection kit (Keygen, Beijing, China) according to the manufacturer’s recommendations. A flow cytometer (ACEA, CA, USA) was used for measuring the cells in sub G1, G1, S, and G2/M phases.

### Phosphatidylserine protein antibody (Annexin V)-FITC/PI assay

The Annexin V-Fluorescein isothiocyanate (FITC) and propidium iodide (PI) apoptosis detection kit (Keygen, Beijing, China) was used to determine cell apoptosis, according to the manufacturer’s instructions. The cells were suspended in 500 μL binding buffer and double-stained with annexin V-FITC/PI for 15 min, and the cell apoptosis was determined using a flow cytometer (ACEA, CA, USA). The laser excitation wavelength was found to be 488 nm; the green signal from annexin V-FITC was observed at a wavelength of 525 nm and the red signal from PI was observed at a wavelength of 620 nm.

### Molecular docking and dynamics

AutoDock vina 1.1.2 software was used to study molecular docking. The NOB CDX format files were prepared by ChemDraw software. The PDB format files for protein structures of p53 (PDB ID: 6GGC) were downloaded from the protein data bank. Both NOB and proteins were processed using the AutoDock tools, and parameterized and saved in the pdbqt format. In terms of docking process, the parameter of exhaustiveness was set as 32, and the other parameters were set as default. The conformation with the highest score was selected as the final docking conformation for subsequent molecular dynamics simulation.

AMBER 18 was used to conduct the molecular dynamics simulation on the complexes generated by molecular docking. The gaff universal force field was used as NOB force field, and the ff14SB force field was used as protein force field. In the tLeap module, adding hydrogen, water and antagonist ion, the topology file and coordinate file were saved for molecular dynamics simulation. The molecular simulation system was conducted a 50 ns production run after energy minimization, heating and equilibration.The generated molecular simulation trajectory was analyzed by the Cpptraj module, and the Molecular mechanics/generalized born surface area (MMGBSA) algorithm was used to calculate the binding free energy of the small molecules.

### UV visible spectroscopy

p53 protein was mixed with the same volume of NOB and maintained for 10 min under 37°C. The final concentration of p53 was 1 μM, and those of NOB were 0, 10, 33, and 100 μM. The absorbance of mixture solution was determined at the wavelength range of 240–700 nm.

### Fluorescence spectroscopy

p53 protein was mixed with the same volume of NOB and maintained for 10 min under 37°C. The final concentration of p53 was 1 μM, and those of NOB were 0, 10, 33, and 100 μM. The fluorescence value of the mixture solution was determined at the wavelength range of 320–440 nm by setting the excitation slit width and the emission slit width as 5 nm, with an excitation wavelength of 280 nm.

### Circular dichroism

The circular dichroism of p53 protein was measured using the J-810 circular dichroism spectrometer (JASCO, Tokyo, Japan). P53 protein was mixed with the same volume of NOB and maintained for 10 min at 37°C. The final concentration of p53 was 1 μM, and those of NOB were 0, 10, 33, and 100 μM. The mixture was scanned in the far-ultraviolet region (198–250 nm) with a scanning rate of 50 nm/min at 37°C. The average residue molecular weight was 115 g/mol, and the proportion of each secondary structure (*α*-helix, *β*-sheet, *β*-turn, and random coil) was analyzed using the program built in the device.

### Statistical analysis

The data were presented as mean ± SD. All experiments were conducted three times independently. One way analysis of variance (one-way ANOVA) was conducted by Statistical Analysis System (SAS Institute Inc., Cary, NC, USA). The Duncan multiple range test was used to determine the differences in mean values between groups (*P* < 0.05). All figures were prepared using GraphPad Prism software (version 6.00, GraphPad Software Inc., San Diego, CA, USA).

## Results

### NOB improved cell viability and cell membrane integrity of JEG-3 and BeWo caused by hypoxia

The CCK-8 assay is a commonly used indicator for evaluating cell viability, representing cell metabolism and mitochondrial activity (16). The degree of damage to the cell membrane can be estimated by detecting the release of LDH, which is a biochemical indicator of the cell membrane integrity (17). The effects of NOB on cell viability and cell membrane integrity of JEG-3 and BeWo were tested by CCK-8 test and LDH assay, respectively. Exposure to hypoxia treatment significantly decreased cell viability and increased LDH activity, compared with those under normoxia treatment (Fig. 2). The treatment of hypoxic cultures with 100 μM NOB significantly increased the cell viability and decreased the LDH activity of JEG-3 and BeWo cells, compared with those exposed to hypoxia treatment. These results revealed that cell viability and cell membrane integrity were decreased by hypoxia, whereas NOB could alleviate this effect.

**Fig. 1:**
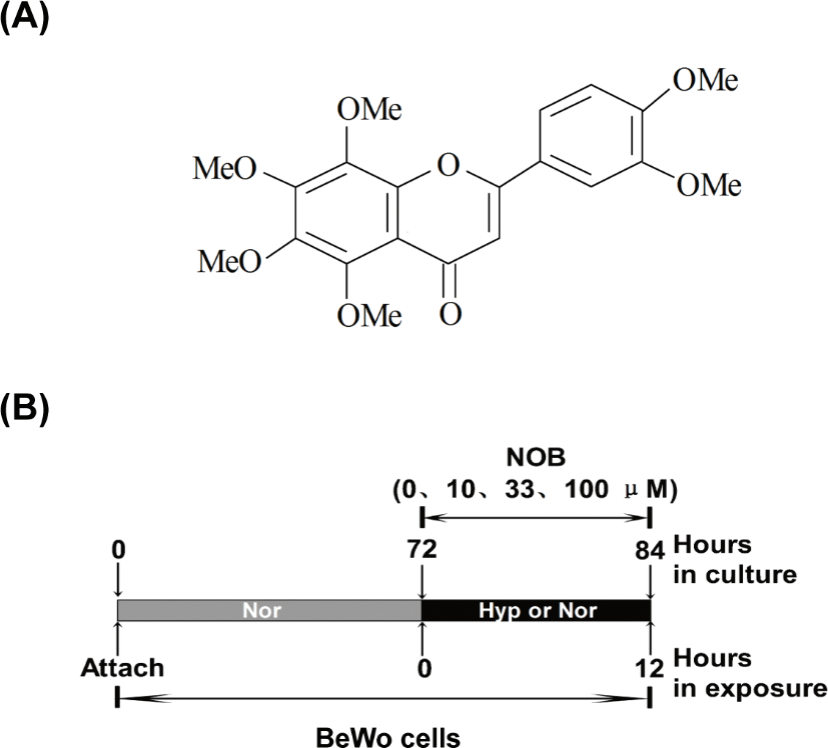
Chemical structure of nobiletin (5,6,7,8,3,4-hexamethoxyflavone, NOB).

**Fig. 2:**
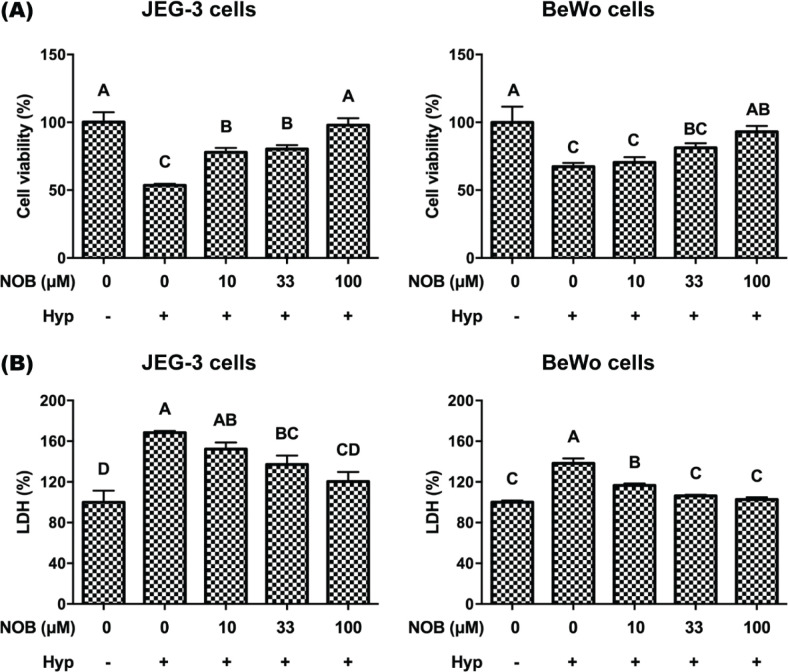
Cell activity and cell membrane integrity of JEG-3 and BeWo were inhibited by hypoxia and could be rescued by NOB. (a) The cell activity was measured using a CCK-8 assay. (b) The cell membrane integrity was measured through the viability of LDH. The JEG-3 and BeWo cells were, respectively, exposed to 0, 10, 33, or 100 μM NOB with 500 μM cobalt chloride in Serum-free medium for 12 h. Cells treated without cobalt chloride and NOB for 12 h were taken as the normoxic control group. The values are presented as the mean ± SD of independent experiments (*n* = 3). All different capital letters indicate significant differences at *P* < 0.05 using one-way ANOVA.

### NOB protected JEG-3 and BeWo cells from biological hypoxia

**Fig. 3:**
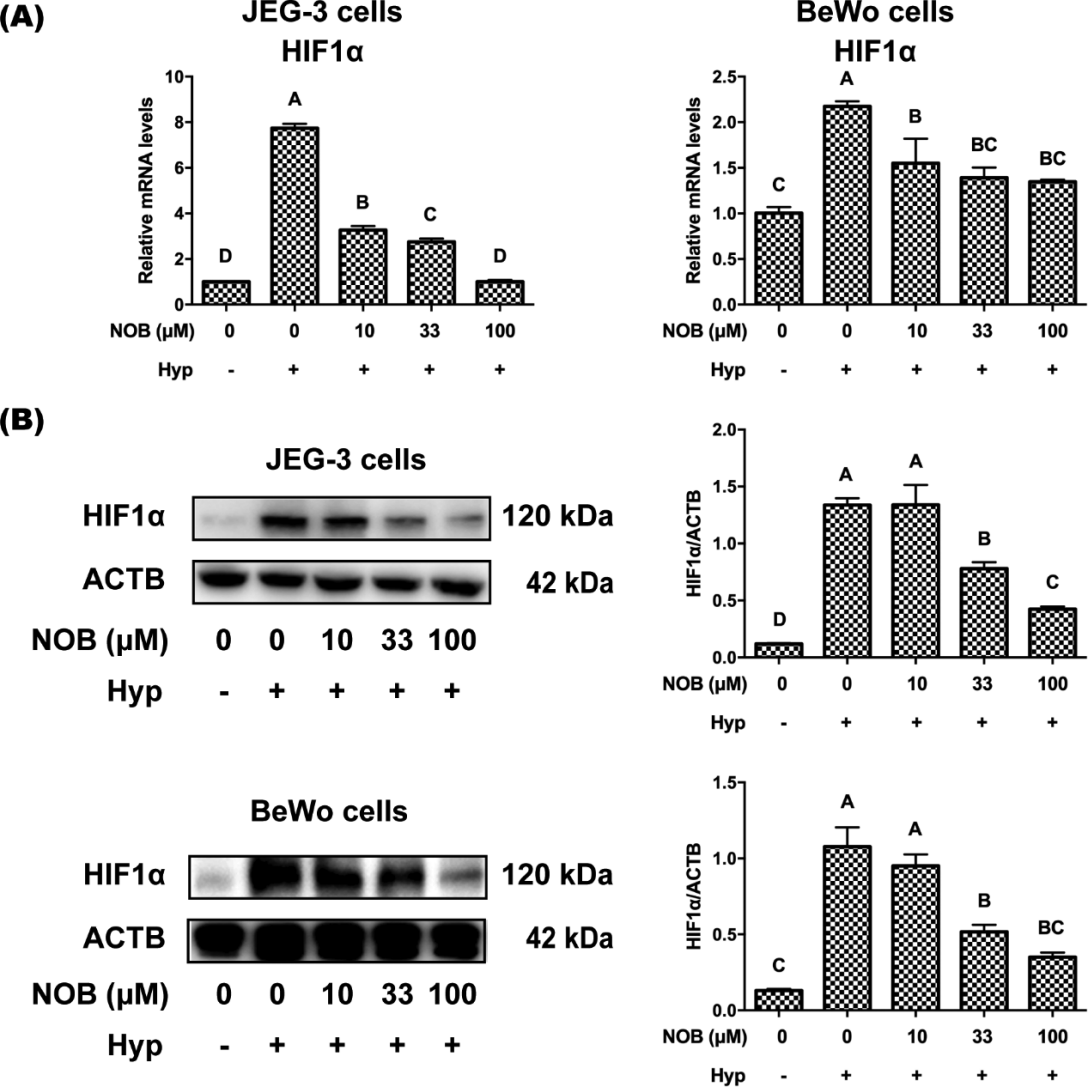
NOB up-regulated the mRNA and protein levels of HIF1α in JEG-3 and BeWo exposed to hypoxia. (a) The mRNA expressions of HIF1α in JEG-3 and BeWo cells were estimated by RT-qPCR analyses. (b) The protein expressions of HIF1α in JEG-3 and BeWo cells were estimated by Western blot analysis. The JEG-3 and BeWo cells were, respectively, exposed to 0, 10, 33, or 100 μM NOB with 500 μM cobalt chloride in Serum-free medium for 12 h. Cells treated without cobalt chloride and NOB for 12 h were taken as the normoxic control group. The values are presented as the mean ± SD of independent experiments (*n* = 3). All different capital letters indicate significant differences at *P* < 0.05 using one-way ANOVA.

HIF1*α* is a sensitive oxygen sensor and a biomarker of hypoxia (18). All mammals control the cellular response to hypoxia by regulating the HIF1*α* transcription factor family (19). To illustrate the effect of NOB on HIF1*α* in hypoxia-induced JEG-3 and BeWo cells, HIF1*α* levels were measured by RT-qPCR and Western blot analysis. HIF1*α* levels were significantly increased in JEG-3 and BeWo cells under hypoxic environment, which was rescued by the treatment of NOB (Fig. 3), suggesting that NOB could protect cells from biological hypoxia.

### NOB reduced G1 phase arrest in hypoxia-induced cells of JEG-3 and BeWo

Cell populations of JEG-3 and BeWo in the G1 phase were significantly arrested, and the number of cells in the S phase was significantly decreased after cells were exposed to hypoxia, compared with those under normoxia (Table 2). For JEG-3 cells, the G1 and S populations were decreased significantly, while the sub G1 and G2/M populations were significantly increased after treatment with 100 μM NOB. With regard to BeWo cells, after incubation with 100 μM NOB, the G1 populations were markedly reduced, while G2/M populations were significantly increased.

**Table 2:** The effect of NOB on cell cycle distribution of hypoxia-induced JEG-3 and BeWo cells

Cell types	Treatment	Sub-G1 (%)	G1 (%)	S (%)	G2/M (%)
JEG-3 cells	NOB 0 μM	1.05 ± 0.19^D^	19.51 ± 1.36^D^	52.08 ± 1.40^A^	27.37 ± 2.31^B^
	Hyp+NOB 0 μM	3.45 ± 0.63^C^	22.66 ± 1.36^A^	45.84 ± 1.42^B^	28.04 ± 1.15^B^
	Hyp+NOB 10 μM	4.74 ± 0.70^B^	22.18 ± 0.90^AB^	45.13 ± 1.81^B^	27.95 ± 1.70^B^
	Hyp+NOB 33 μM	4.18 ± 0.68^BC^	21.40 ± 0.51^BC^	46.44 ± 1.52^B^	27.98 ± 0.84^B^
	Hyp+NOB 100 μM	6.92 ± 2.05^A^	20.37 ± 1.79^C^	43.05 ± 1.78^C^	29.67 ± 1.42^A^
	NOB 0 μM	1.86 ± 0.60^B^	25.52 ± 1.19^C^	55.51 ± 0.84^A^	17.10 ± 0.80^BC^
BeWo cells	Hyp+NOB 0 μM	1.22 ± 0.22^B^	40.08 ± 1.67^A^	43.55 ± 1.47^C^	15.14 ± 0.91^C^
	Hyp+NOB 10 μM	5.53 ± 1.83^A^	28.69 ± 3.33^C^	42.67 ± 1.97^C^	23.11 ± 4.24^A^
	Hyp+NOB 33 μM	1.13 ± 0.10^B^	34.20 ± 0.48^B^	46.86 ± 1.99^B^	17.83 ± 1.63^BC^
	Hyp+NOB 100 μM	1.45 ± 0.17^B^	33.24 ± 1.18^B^	43.78 ± 1.37^C^	21.54 ± 2.41^AB^

The cell cycle distribution of JEG-3 and BeWo cells was detected by flow cytometry with PI staining. The JEG-3 and BeWo cells were, respectively, exposed to 0, 10, 33, or 100 μM NOB with 500 μM cobalt chloride in Serum-free medium for 12 h. Cells treated without cobalt chloride and NOB for 12 h were taken as the normoxic control group. The values are presented as the mean ± SD of independent experiments (*n* = 3). For a specific cell cycle distribution (Sub-G1, G1, S, or G2) in the same cell lines, all different capital letters indicate significant differences at *P* < 0.05 using one-way ANOVA.

### NOB attenuated hypoxia-induced apoptosis in JEG-3 and BeWo cells

**Fig. 4:**
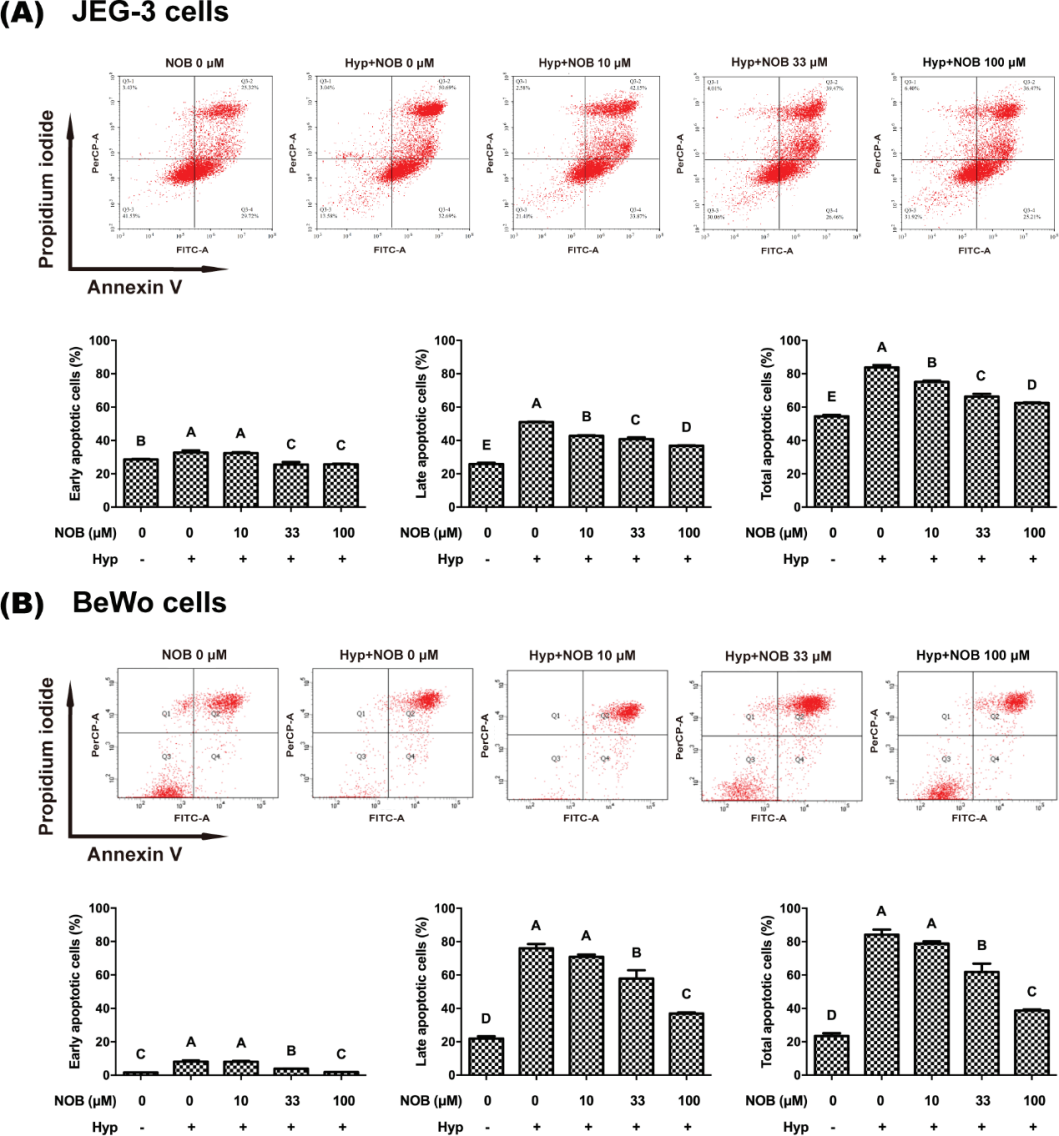
NOB attenuated hypoxia-mediated apoptosis in JEG-3 and BeWo cells. The apoptosis of JEG-3 and BeWo cells was detected by flow cytometry with Annexin V and PI double staining. (a) The apoptosis degrees of JEG-3 cells. (b) The apoptosis degrees of BeWo cells. The JEG-3 and BeWo cells were, respectively, exposed to 0, 10, 33, or 100 μM NOB with 500 μM cobalt chloride in Serum-free medium for 12 h. Cells treated without cobalt chloride and NOB for 12 h were taken as the normoxic control group. The values are presented as the mean ± SD of independent experiments (*n* = 3). All different capital letters indicate significant differences at *P* < 0.05 using one-way ANOVA.

To study whether NOB could attenuate hypoxia-induced apoptosis in JEG-3 and BeWo cells, flow cytometric analysis of annexin V or PI double staining was performed by detecting the externalization of phosphatidylserine in the membrane of early apoptotic cells (annexin positive and PI negative) and the loss of cell membrane integrity in late apoptotic cells (annexin positive and PI positive) (20). For JEG-3 and BeWo cells, the early, late, and total apoptotic cells increased significantly in cells under hypoxic environment compared with those under normoxia (Fig. 4). The apoptosis of hypoxia-induced cells was alleviated by treating with NOB at the concentrations of 33 and 100 μM.

**Fig. 5:**
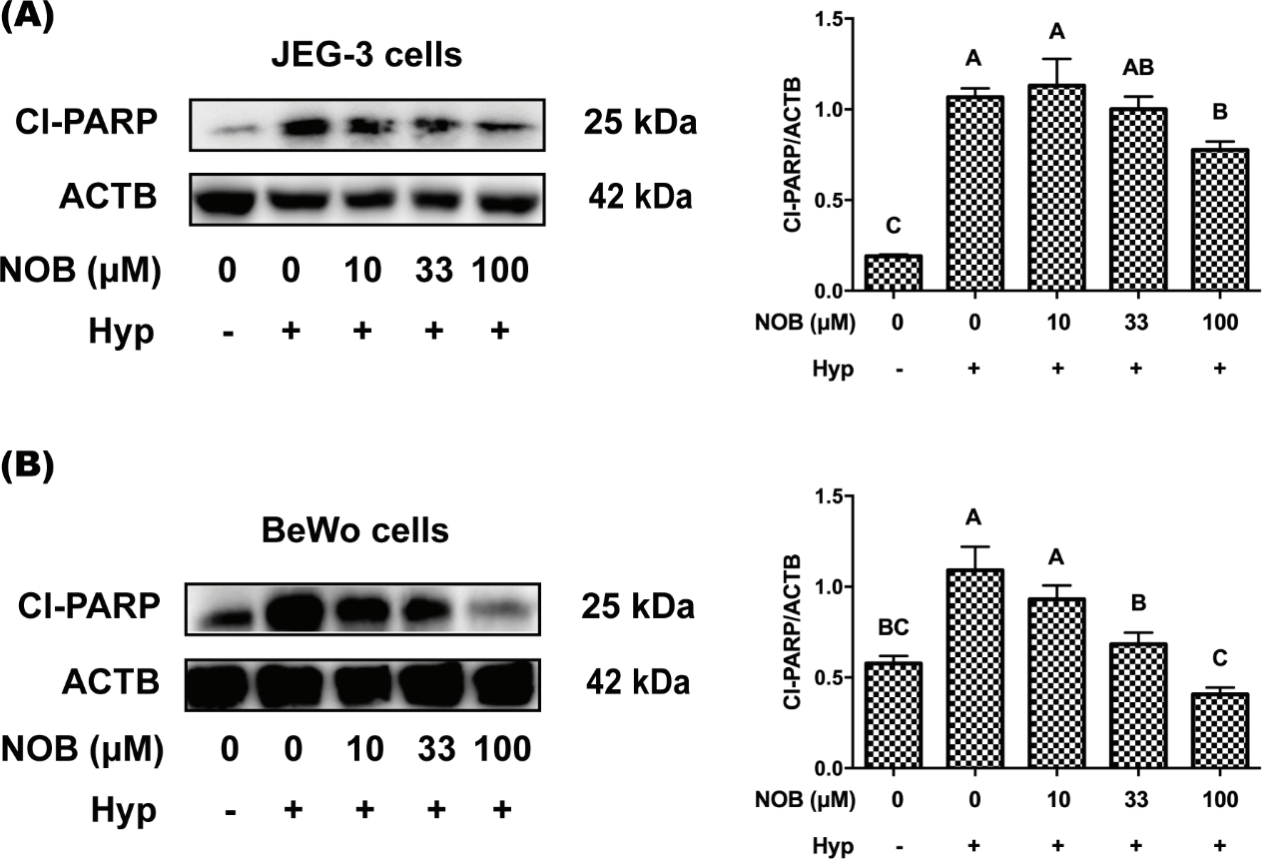
The effect of NOB on apoptosis marker protein of hypoxia-induced JEG-3 and BeWo cells. The expression of cleaved PARP (cl-PARP) was estimated by Western blot analysis. (a) The cl-PARP levels of JEG-3 cells. (b) The cl-PARP levels of BeWo cells. The JEG-3 and BeWo cells were, respectively, exposed to 0, 10, 33, or 100 μM NOB with 500 μM cobalt chloride in Serum-free medium for 12 h. Cells treated without cobalt chloride and NOB for 12 h were taken as the normoxic control group. The values are presented as the mean ± SD of independent experiments (*n* = 3). All different capital letters indicate significant differences at *P* < 0.05 using one-way ANOVA.

Moreover, PARP is an enzyme used for DNA repair and a major substrate for caspase-3 (21). Once PARP is cleaved, it loses enzyme activity and its ability to repair DNA (22). Therefore, the cleaved PARP (cl-PARP) level was detected by Western blot analysis to characterize the effect of NOB on caspase-induced apoptosis. As presented in Fig. 5, compared with the cells under normoxia, the level of cl-PARP was statistically increased in hypoxia-induced cells of JEG-3 and BeWo. The cl-PARP of cells treated with 100 μM NOB was reduced compared with those exposed to hypoxia treatment. All of the above results revealed that NOB at 100 μM concentration could reduce the apoptosis of BeWo and JEG-3 cells induced by hypoxia.

### NOB and p53 protein combined stably with van der Waals force

**Fig. 6:**
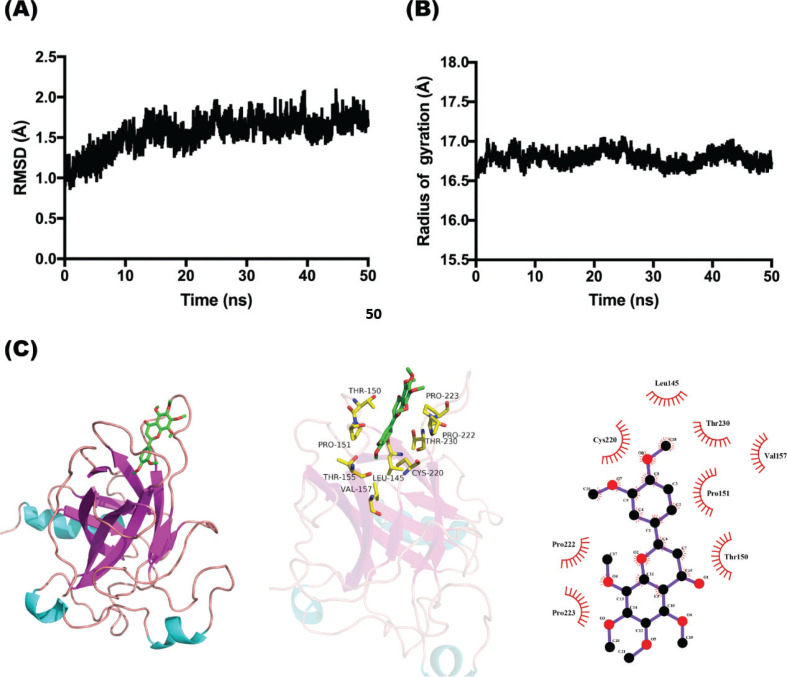
Molecular docking and dynamics and of NOB and p53 protein. (a) The time dependence of root mean square deviation (RMSD) between NOB and p53 protein by molecular dynamic. (b) The time evolution of the radius of gyration (Rg) between NOB and p53 protein by molecular dynamic. (c) The molecular docking of NOB and p53 protein.

P53, a major cancer suppressor gene, regulates apoptosis by activating the p53 pathway in response to DNA damage induced by hypoxia (23). The simulation time of molecular dynamics simulation to study protein structure changes is usually 10–50 ns (24). In this study, the molecular dynamics model with a simulation time of 50 ns was selected to analyze the interaction between NOB and p53 protein. The root mean square deviation (RMSD) is the average deviation between the protein conformation at a specific time and the original structure, which is an important basis for measuring the stability of the system (25). As shown in Fig. 6a, in the first 25 ns of the simulation, the RMSD fluctuates greatly, and after 25 ns, it stabilizes. NOB bonded well to the p53 protein, and the binding of NOB was conducive to the stable existence of the p53 protein, with less conformational fluctuations. The radius of gyration (Rg) characterizes the tightness of protein structure (26). As shown in Fig. 6b, during the dynamics simulation process, Rg was stable without major fluctuations, and the p53 protein structure remained compact throughout.

Furthermore, the binding free energy was calculated for the 25-50 ns (after dynamics trajectory equilibrium) conformation, and the results are shown in Table 3. NOB and p53 protein bonded well, and its binding free energy was estimated to be −26.4 ± 3.38 kcal/mol; the essential driving force involved was van der Waals interactions. The view of the interaction between NOB and p53 protein after 50 ns dynamics simulation is shown in Fig. 6c. NOB interacted with Leu145, Thr150, Pro151, Thr155, Val157, Cys220, Pro222, Pro223, and Thr230 in the flexible ring region of p53.

**Table 3:** Docking energies of NOB with p53 protein

Energies of NOB with p53 protein	
Van der Waals potential energy	-35.61 ± 3.54 Kcal.mol^-1^
Electrostatic interaction	-1.44 ± 3.75 Kcal.mol^-1^
Polarization solvation	15.08 ± 3.44 Kcal.mol^-1^
Non-polarized solvation	-4.38 ± 0.35 Kcal.mol^-1^
Gas phase binding energy	-37.06 ± 5.41 Kcal.mol^-1^
Solvation free energy	10.70 ± 3.31 Kcal.mol^-1^
Combined free energy	-26.36± 3.38 Kcal.mol^-1^

### The interaction between NOB and p53 protein was further verified in vitro

#### UV-visible spectrum of NOB and p53 protein

**Fig. 7:**
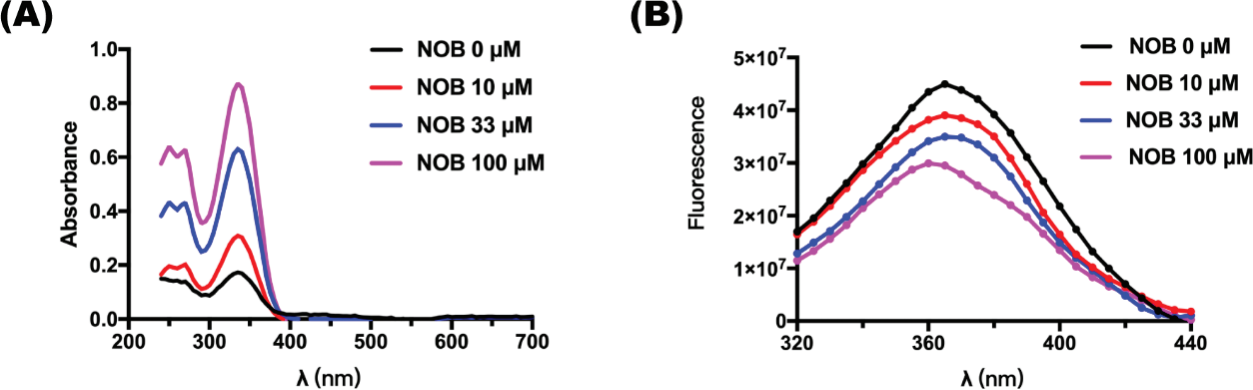
The interaction between NOB and p53 protein was verified *in vitro*. (a) UV-visible spectrum of the interaction between NOB and p53 protein. (b) Fluorescence spectrum of the interaction between NOB and p53 protein. Experimental conditions: 37°C, p53 protein was mixed with the same volume of NOB and maintained for 10 min. The final concentration of p53 was 1 μM, and those of NOB were 0, 10, 33, or 100 μM.

The UV-visible spectrum was used to detect whether the ligand was bonded to the protein during the formation of the compound, and the changing value reflexed the change of the secondary protein structure (27). Figure 7a shows that the compound of NOB and p53 protein had three absorption peaks: 250, 270, and 335 nm. With the increase of NOB concentration, the absorption peak rose correspondingly, indicating that NOB changed the secondary structure of p53 protein.

### Fluorescence spectroscopy of NOB and p53 protein

The amino acid residue of tryptophan, tyrosine, and phenylalanine in p53 protein emits intrinsic fluorescence when excited at a wavelength of 280 nm. Figure 7b shows the fluorescence quenching of p53 protein with the NOB at different concentrations. With the increase of NOB concentration, the intensity of the intrinsic fluorescence for p53 protein gradually decreased, with the blue shift of peaks (Δλ = 5 nm), indicating that NOB entered the hydrophobic pocket of p53 protein and interacted with the protein, and therefore, the hydrophobicity of the p53 protein increased. To investigate the mechanism of fluorescence quenching, we calculated the quenching constant following the Stern-Volmer formula (28):

$$\frac{F_{0}}{F}=1+K_{SV}\left[Q\right]=1+K_{q}\tau_{0}\left[Q\right],$$ (1)

where F_0_ is the fluorescence intensity of free p53 acid (a.u.), F is the fluorescence intensity of p53 protein after the treatment of NOB (a.u.), *Ksv* is the quenching rate constant, [*Q*] is the NOB concentration (mol·L^-1^), *Kq* is the bimolecular quenching rate constant (L·mol^-1^·s^-1^), and τ0 is the average lifetime of the fluorescent molecules in the absence of a quencher. the fluorescence lifetime of biomacromolecules is about 10^-8^ s.

The calculated quenching rate constant (*Ksv*) was 2.1 × 10^3^ L·mol^-1^, and *Kq* was 2.1 × 10^11^ L·mol^-1^·s^-1^ (Table 4), which is much greater than 2.0 × 10^10^ L·mol^-1^·s^-1^, the maximum quenching constant of biomacromolecules (29). Hence, it was determined that the quenching mechanism was static quenching. It is also suggested that NOB entered the hydrophobic pocket of p53 protein, and the interaction changed the p53 protein conformation, finally leading to the quenching of p53 protein fluorescence.

**Table 4:** The binding constant of NOB and p53 protein

*T* (°C)	*K_SV_* (L·mol^-1^)	*Kq*( L·mol^-1^·s^-1^)	*R* ^2^
37	2.10 × 10^3^	2.10 × 10^11^	0.96

*Ksv* is the quenching rate constant and *Kq* is the bimolecular quenching rate constant (L·mol^-1^·s^-1^).

### Circular dichroism of NOB and p53 protein

Circular dichroism is an excellent method for rapid determination of the secondary protein structures (30). Compared with the non-NOB-treated group, the α-helix and random coil of p53 protein were decreased, and the β-sheet was increased after treatment of NOB at 100 μM (Table 5). The results revealed that the interaction of NOB and p53 protein led to transformation of the α-helix and random coil of p53 protein into β-sheet.

**Table 5:** Secondary structure of p53 protein adding various NOB

NOB	α-helix	β-sheet	β-turn	Random coil
0 μM	10.95 ± 0.35^A^	25.85 ± 0.35^B^	33.85 ± 0.49^B^	29.35 ± 0.49^A^
10 μM	8.00 ± 0.42^B^	26.65 ± 1.20^B^	37.40 ± 1.41^A^	28.00 ± 0.14^B^
33 μM	7.35 ± 0.35^BC^	28.80 ± 0.57^AB^	36.55 ± 0.78^A^	27.25 ± 0.21^B^
100 μM	6.45 ± 0.64^C^	30.25 ± 2.05^A^	36.30 ± 0.85^AB^	27.00 ± 0.57^B^

Secondary structures of p53 protein were detected by circular dichroism. Under 37°C, p53 protein was mixed with the same volume of NOB and maintained for 10 min. The final concentration of p53 was 1 μM, and those of NOB were 0, 10, 33, or 100 μM. The values are presented as the mean ± SD of independent experiments (*n* = 3). In the same column, all different capital letters indicate significant differences at *P* < 0.05 using one-way ANOVA.

### Regulation of NOB on p53 in hypoxia-induced cells of JEG-3 and BeWo

**Fig. 8:**
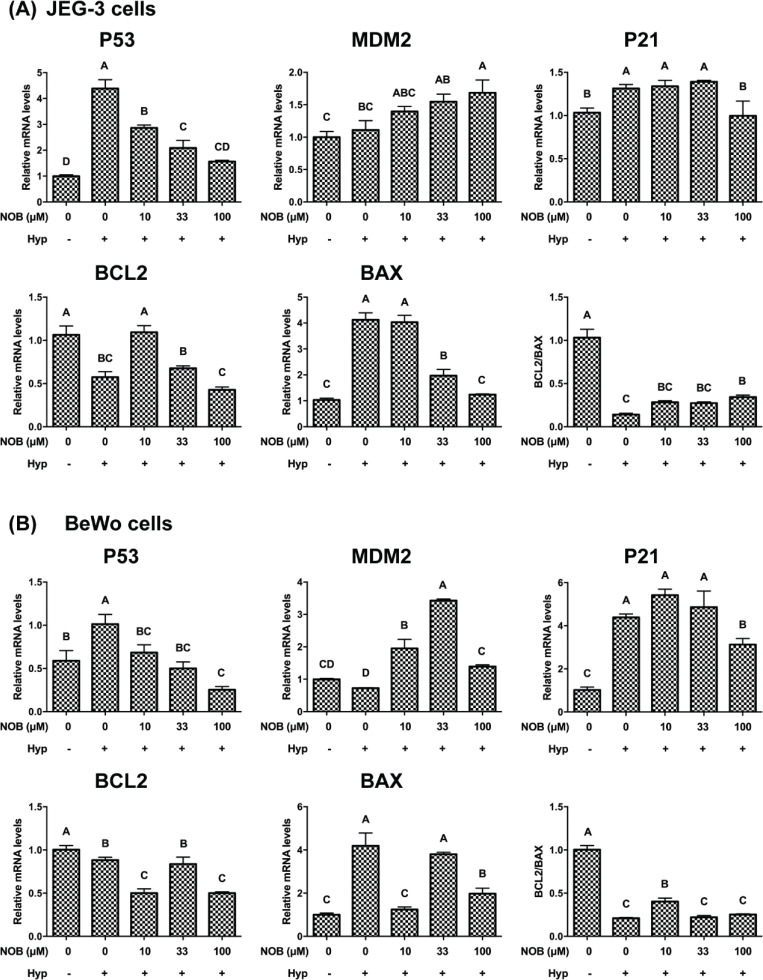
The effect of NOB on expressions of p53 pathway genes of hypoxia-induced JEG-3 and BeWo cells. The mRNA expressions of p53, MDM2, p21, BCL2, and BAX were estimated by RT-qPCR. The mRNA ratio of BCL2/BAX was calculated. (a) The mRNA expression analysis of JEG-3 cells. (b) The mRNA expression analysis of BeWo cells. The JEG-3 and BeWo cells were, respectively, exposed to 0, 10, 33, or 100 μM NOB with 500 μM cobalt chloride in Serum-free medium for 12 h. Cells treated without cobalt chloride and NOB for 12 h were taken as the normoxic control group. The values are presented as the mean ± SD of independent experiments (*n* = 3). All different capital letters indicate significant differences at *P* < 0.05 using one-way ANOVA.

**Fig. 9:**
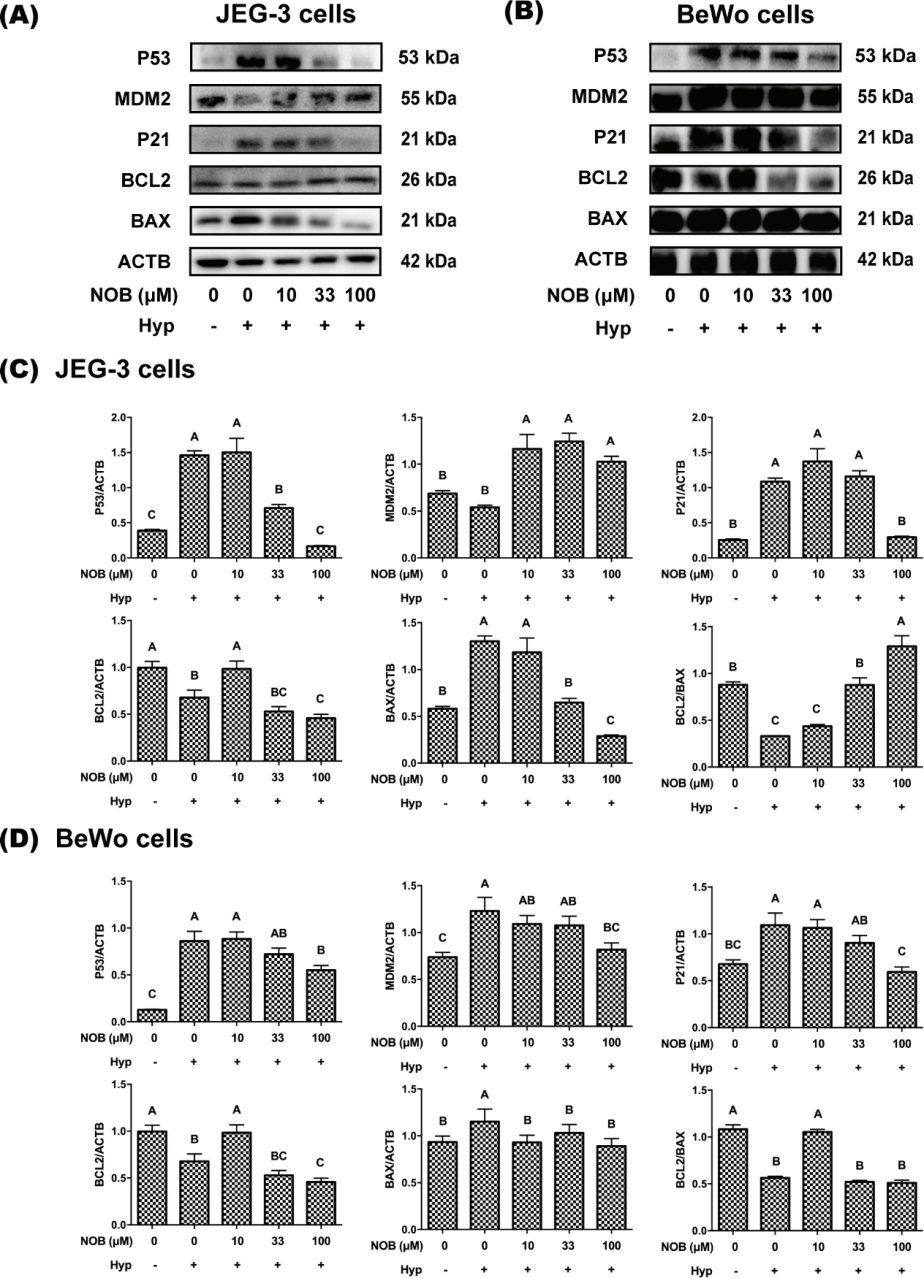
The effect of NOB on expressions of p53 pathway proteins of hypoxia-induced JEG-3 and BeWo cells. The protein expressions of p53, MDM2, p21, BCL2 and BAX were estimated by Western blot analysis. The protein ratio of BCL2/BAX was calculated. (a) The Western blot of JEG-3 cells. (b) The Western blot of BeWo cells. (c) The protein expression analysis of JEG-3 cells. (d) The protein expression analysis of BeWo cells. The JEG-3 and BeWo cells were, respectively, exposed to 0, 10, 33, or 100 μM NOB with 500 μM cobalt chloride in Serum-free medium for 12 h. Cells treated without cobalt chloride and NOB for 12 h were taken as the normoxic control group. The values are presented as the mean ± SD of independent experiments (*n* = 3). All different capital letters indicate significant differences at *P* < 0.05 using one-way ANOVA.

To investigate the regulation of p53 on apoptosis, p53 and its related genes and proteins were measured by RT-qPCR and Western blot analysis. Compared with the cells under normoxia, the mRNA and protein levels of p53, p21, and BAX were increased in JEG-3 and BeWo cells under the hypoxic environment, while the mRNA and protein levels of BCL2 and the ratio of BCL2/BAX were decreased (Figs. 8, 9). Treatment of the JEG-3 cells with NOB at a concentration of 100 μM significantly reduced the mRNA and protein levels of p53, p21, and BAX, and significantly increased the mRNA and protein ratio of BCL2/BAX in hypoxia-induced cells. As for BeWo cells, the mRNA and protein levels of p53 and p21 were decreased after hypoxia-induced cells were treated with 100 μM NOB, while no differences in the mRNA and protein ratio of BCL2/BAX were observed. These findings suggested that p53 was involved in the regulation of apoptosis by NOB.

## Discussion

With the development of the food industry, NOB has been developed and used as a natural phytochemical. This research study revealed the anti-apoptotic effect of NOB, which was observed in hypoxia-induced JEG-3 and BeWo cells, as measured by annexin V-FITC/PI and cl-PARP assays. With the treatment of 100 μΜ NOB, there was a decrease in the mRNA and protein levels of HIF1α and the G1 phase arrest. NOB and human p53 protein bonded spontaneously, leading to change in the conformation of p53 protein, which was detected by molecular docking and dynamics. These findings were also proved *in vitro* by UV-visible spectroscopy, fluorescence spectroscopy, and circular dichroism. JEG-3 and BeWo cells treated with 100 μΜ NOB down-regulated the mRNA and protein expressions of p53 and p21. The mRNA and protein ratio of BCL2/BAX in hypoxia-induced JEG-3 cells was up-regulated after treatment with 100 μM NOB, but did not change the BCL2/BAX ratio for BeWo cells.

HIF1α activation is the most recognized response to hypoxia in mammalian cells, and its activity can be stabilized by CoCl_2_ to prevent degradation by pan-hydroxylase (19). After stabilization, HIF1α and its cofactors bind to the HIF response element in the promoters of the target genes to coordinate an extensive transcriptional program targeting the hypoxic environment (31). Annexin V-FITC/PI, a phenotypic indicator of apoptosis, proved that JEG-3 and BeWo cells had undergone apoptosis. The apoptosis marker protein of cl-PARP was also activated during this process. These results suggested that a hypoxia-induced apoptosis model was successfully established by CoCl_2_ in this study.

Many phytochemicals have protective effects on hypoxia-induced trophoblast cell apoptosis. Rosiglitazone was found to inhibit the activity of caspase-3 and caspase-9, as well as to reduce the TUNEL positive rate in JEG-3 and BeWo cells cultured in 2% O_2_ (4).Punicalagin was shown to downregulate p53 and attenuate apoptosis in human placental STB in 1% O_2_ (32). Furthermore, melatonin has been reported to inhibit apoptosis because it was observed a decline of cl-PARP level in human placental STB undergo hypoxia (1% O_2_)/reoxygenation (8% O_2_). Many phytochemicals, such as quercetin, herpersin, and hesperetin, have shown anti-apoptotic effects in HTR-8/SVneo cells induced by hypoxia (0.2% O_2_)/reoxygenation (95% air, 5% CO_2_) by inhibiting the activities of caspase-3 and caspase-7 (34). NOB, a kind of flavonoid containing six methoxy groups, has been reported to reduce cardiomyocyte apoptosis in mice with myocardial hypertrophy (9). This study established that NOB reduced the sensitivity of JEG-3 and BeWo cells in response to hypoxia, which was manifested by the decrease in HIF1α levels, and in turn, reduced apoptosis.

The p53 tumor suppressor protein plays an important role in the monitoring of hypoxic stress signals by activating specific transcriptional targets that control the cell cycle arrest and apoptosis (22, 25, 35). Molecular docking and dynamics simulations showed that NOB and p53 protein bond spontaneously, and van der Waals potential energy was its main driving force. When binding to NOB, the conformations of p53 protein tended to be stable after small fluctuations, and the structures kept compact throughout. It was verified by UV-visible spectroscopy that NOB modified the secondary structure of p53 protein *in vitro*. This was because NOB entered the hydrophobic pocket of p53 protein and caused static quenching of p53 protein, which was proved by fluorescence spectroscopy. Circular dichroism spectroscopy further confirmed that the interaction of NOB and p53 protein led to the transformation of the secondary structure of p53 protein, α-helix and random coil, into β-sheet. These results suggested that the entry of NOB into cells may affect the function and activity of p53 protein. The post-transcriptional and post-translational modifications of p53 protein have an impact on downstream specific transcription targets.

P53 is accumulated with the activation of HIF1α in the hypoxic region (36), as transcriptionally active p53 is stabilized through a physical association with HIF1α (37), and the direct binding between HIF1α and MDM2 suppressed MDM2-dependent ubiquitylation of p53 *in viv*o and p53 nuclear export (38). P21, a downstream gene of p53, is required for p53-mediated G1 and G2 cell cycle arrest, of which p21 is more effective in preventing G1 progression (39). If hypoxia-induced DNA damage accumulation is severe or unrepairable, the failure of cells to exit the cell cycle from incomplete mitosis may lead to induction of apoptosis (40). Many natural products, especially those from medicinal and food plants, have been reported to target p53 to inhibit apoptosis. Punicalagin was found to attenuate hypoxia-induced apoptosis by reducing p53 and HIF1α levels in cultured human placental STB (32). Curcumin and silymarin were shown to inhibit paracetamol-induced hepatocellular apoptosis by observing an apparent increase in the number of p53-stained cells in adult male albino rats (41). Lycium barbarum polysaccharides were reported to protect H9c2 cells from hypoxia-induced apoptosis by down-regulation of p53 (42). Glucomoringin isothiocyanate was found to reduce apoptosis in H_2_O_2_-induced SHSY5Y cells by inhibiting p53 activity (43). In this study, p53 was involved in the regulation of apoptosis by NOB, which was proved by the decrease of expression levels of p53 and p21, as well as cell cycle arrest in the G1 phase.

P53 is entirely nuclear, and it is certainly conceivable that a portion of hypoxia stabilized p53 translocates from the nucleus to the cytoplasm (44). BAX and Sival are transcriptional target genes of p53, and Sival can reduce the levels of downstream BCL2 and BCLXL. Therefore, the balance between anti-apoptotic and pro-apoptotic members of BCL2 family can be regulated by p53 to affect the outcome of mitochondrial-dependent intrinsic apoptosis. These apoptotic effects were neutralized by the formation of heterodimeric complexes between BAK and BCL2 proteins (45). The literature has shown the anti-apoptotic activities of some phytochemicals through the intrinsic apoptosis pathway. Apoptosis in IND-induced GES-1 cells was inhibited by total triterpenoids through the up-regulation of BCL2, BCLXL, and BCL2/BAX ratio (46). Apoptosis of Aβ-induced PC12 cells was attenuated after the treatment of schisandrin and nootkatone by increasing the level of Bcl-2 and decreasing the levels of BAD and BAX (47). Mitochondria-mediated apoptosis was decreased by the treatment of 5-heptadecylresorcinol, which increased the BCL2/BAX ratio in neurocytes (48). Acrylamide-induced neuronal apoptosis was attenuated by the treatment of garcinol, which reduced the BCL2/BAX ratio in the brain of zebrafish larvae (49). This study found that 100 μM NOB increased the ratio of BCL2/BAX in JEG-3 cells, but no change was observed in BeWo cells, suggesting that NOB might protect JEG-3 cells against hypoxia by the p53-mediated intrinsic apoptosis pathway, and the anti-apoptotic effect of NOB might be due to other p53-mediated pathways, such as external apoptotic pathways in BeWo cells.

## Conclusions

The G1 cell cycle arrest and apoptosis induced by hypoxia were attenuated by the treatment of 100 μM NOB in JEG-3 and BeWo cells. With regard to JEG-3 cells, NOB protected JEG-3 cells from hypoxia through the p53-mediated intrinsic apoptosis pathway. For BeWo cells, this anti-apoptotic effect was specific to the p53, while the intrinsic apoptosis pathway was not involved. These findings suggested that NOB was an effective natural product with promising development to prevent hypoxia-induced apoptosis in trophoblast cells.
